# Cowpea Mosaic Virus Nanoparticle Enhancement of Hypofractionated Radiation in a B16 Murine Melanoma Model

**DOI:** 10.3389/fonc.2020.594614

**Published:** 2020-12-16

**Authors:** Kayla E. A. Duval, Robert J. Wagner, Veronique Beiss, Steven N. Fiering, Nicole F. Steinmetz, P. Jack Hoopes

**Affiliations:** ^1^ Geisel School of Medicine, Dartmouth College, Hanover, NH, United States; ^2^ Thayer School of Engineering, Dartmouth College, Hanover, NH, United States; ^3^ Department of NanoEngineering, University of California San Diego, La Jolla, CA, United States; ^4^ Norris Cotton Cancer Center, Dartmouth-Hitchcock Medical Center, Lebanon, NH, United States; ^5^ Department of Bioengineering, University of California San Diego, La Jolla, CA, United States; ^6^ Department of Radiology, University of California San Diego, La Jolla, CA, United States; ^7^ Moores Cancer Center, University of California San Diego, La Jolla, CA, United States; ^8^ Center for Nano-ImmunoEngineering, University of California San Diego, La Jolla, CA, United States

**Keywords:** radiation, immune response, immunology, cowpea mosaic virus, viral nanoparticles

## Abstract

**Introduction:**

Virus and virus-like nanoparticles (VNPs) have been used for a variety of preclinical treatments, including *in situ* anti-cancer vaccination. The Cowpea mosaic virus (CPMV) is a VNP that has shown the ability to stimulate an anti-cancer immune response. The hypothesis of this study is two-fold: that intratumoral CPMV enhances the immunogenetic and cytotoxic response of hypofractionated radiation (15 Gy or 3 x 8 Gy), and that the effect differs between fraction regimens in the murine B16 flank melanoma model.

**Methods:**

CPMV nanoparticles were delivered intratumorally, 100 μg/tumor to B16 murine melanoma flank tumors alone, and in combination with either 15 Gy or 3 x 8 Gy (3 consecutive days). Tumors were assessed for immune and cytotoxic gene and protein expression, and cytotoxic T cell infiltration 4 days post treatment. Treatment based tumor control was assessed by a 3-fold tumor growth assay.

**Results:**

Both CPMV and radiation alone demonstrated the activation of a number of important immune and cytotoxic genes including natural killer cell and T cell mediated cytotoxicity pathways. However, the combination treatment activated greater expression than either treatment alone. CPMV combined with a single dose of 15 Gy demonstrated greater immune and cytotoxic gene expression, protein expression, CD8+ T cell infiltration activity, and greater tumor growth delay compared to 3 x 8 Gy with CPMV.

**Conclusion:**

CPMV presents a unique and promising hypofractionated radiation adjuvant that leads to increased anti-tumor cytotoxic and immune signaling, especially with respect to the immune mediated cytotoxicity, immune signaling, and toll-like receptor signaling pathways. This improvement was greater with a single dose than with a fractionated dose.

## Introduction

As cancer therapy continues to evolve, immunotherapy has become one of most promising and researched treatment modalities. There are a number of immunotherapy mechanisms, however the general concept in cancer therapy is to manipulate and/or train the patient’s immune system to directly kill cancer cells, block immune suppressive factors, or enhance other therapies. One novel area of immunotherapy is based on the use of virus and virus-like nanoparticles (VNPs) that target and enhance specific anti-cancer immune reactions (1). It is generally believed that this mechanism relies on pattern recognition and initiates the most effective anti-tumor response when delivered directly to the tumor (2, 3). Some investigators have characterized this modality as an anti-cancer VNP *in situ* vaccine (4). One such agent is a plant virus, Cowpea mosaic virus (CPMV). In addition to being a well characterized and researched agent, for several non-cancer biotechnologies, CPMV has recently shown potential as a safe anti-cancer immunotherapy (5, 6). Specifically, CPMV VNPs have demonstrated the ability to improve pre-clinical outcomes by overcoming tumor based immune suppression in local tumor situations (7–9). Additionally, systemically administered CPMV have the ability to stimulate an additive therapeutic immune response following treatment of the tumor with a conventional agent such as radiation (8). With an understanding that ionizing radiation is one of the most effective cancer treatments, and that a significant number of patients remain uncured, it makes sense to combine radiation with other therapies. We are now beginning to understand the positive immune benefits of radiation, especially hypofractionated radiation (HFRT), with larger doses per fraction and fewer fractions than conventional radiation therapy (10). Thus, combining HFRT with immunoactive agents such as CPMV could enhance the overall therapeutic effect. In particular, we hypothesize the combination will lead to a larger cytotoxic immune response, ultimately improving efficacy *via* up-regulation of apoptotic pathways and immune cell activation, both cytotoxic natural killer cells and cytotoxic T cells. Additionally, we hypothesized that the effect would differ with varying fraction regimens. In this study, two hypofractionated radiation doses are used, 3 x 8 Gy, and a single dose of 15 Gy. Biologically effective dose calculations (BED), and the biologically equivalent dose in 2 Gy fractions (EQD2) calculations, indicate that these two radiation schemes are biologically similar.

Our goal was to answer a simple but important question: how do different radiation fraction schemes, when combined with a biocompatible immunogenic plant virus, affect the anti-tumor immunogenetic response. We used 4 straightforward research techniques to address the question, NanoString genetic assessment (cancer immune panel), IHC for CD-8, western blot protein analysis and a mouse tumor regrowth analysis. It is clear that abscopal/metastatic experiments, or immune cell depletion studies, would provide additional and important information. However, those aspect of radiation based immune stimulation were not the intended topic of this research. Clearly, a more comprehensive genetic and IHC immune cell type assessment would have been helpful in a global sense, however, we feel a foundational study, such as this, accurately depicts the promise and potential of hypofractionated radiation combined with immunotherapy.

## Methods

### Cell Line

B16-F10 murine melanoma cells were obtained from the American Type Culture Collection (ATCC), Manassas, VA. Cells were cultured in 1X Roswell Park Memorial Institute (RPMI) with 4.5 g/L glucose and L-glutamine, 10% Fetal bovine serum (FBS), 1% L-Glutamine (200 mM in 0.85% NaCl, HyClone Laboratories, Inc., Logan, UT), and 1% penicillin—streptomycin solution (10,000 units/ml penicillin; 10,000 µg/ml streptomycin, HyClone Laboratories, Inc., Logan, UT). Cells were cultured on tissue culture dishes and incubated at 37°C and 5% CO_2_.

### Mouse Model

B16-F10 tumors were grown intradermally in the right flank region of six-week-old C57BL6 female mice. Animals were placed on study when their tumors reached 100+/-20 mm^3^. The treatment groups (n=8–10) included control, CPMV, 15 Gy, 3x8 Gy, 15 Gy + CPMV, and 3x8 Gy + CPMV. Two experimental time endpoints were used (all groups were assessed at each endpoint). The 4 day post-treatment endpoint (final day of treatment) was used to collect RNA (for genetic assessment), protein, and tissue for CD8^+^ immunohistochemistry (IHC). For the efficacy cohort, the animals were taken off study when a tumor reached 3-times the volume it had on the day of treatment. Tumors were measured three dimensionally using calipers, with volume calculated as length*width*depth*pi/6. These studies were approved by the Dartmouth College Institutional Animal Care and Use Committee (IACUC).

### CPMV Nanoparticles

**Figure 1:**
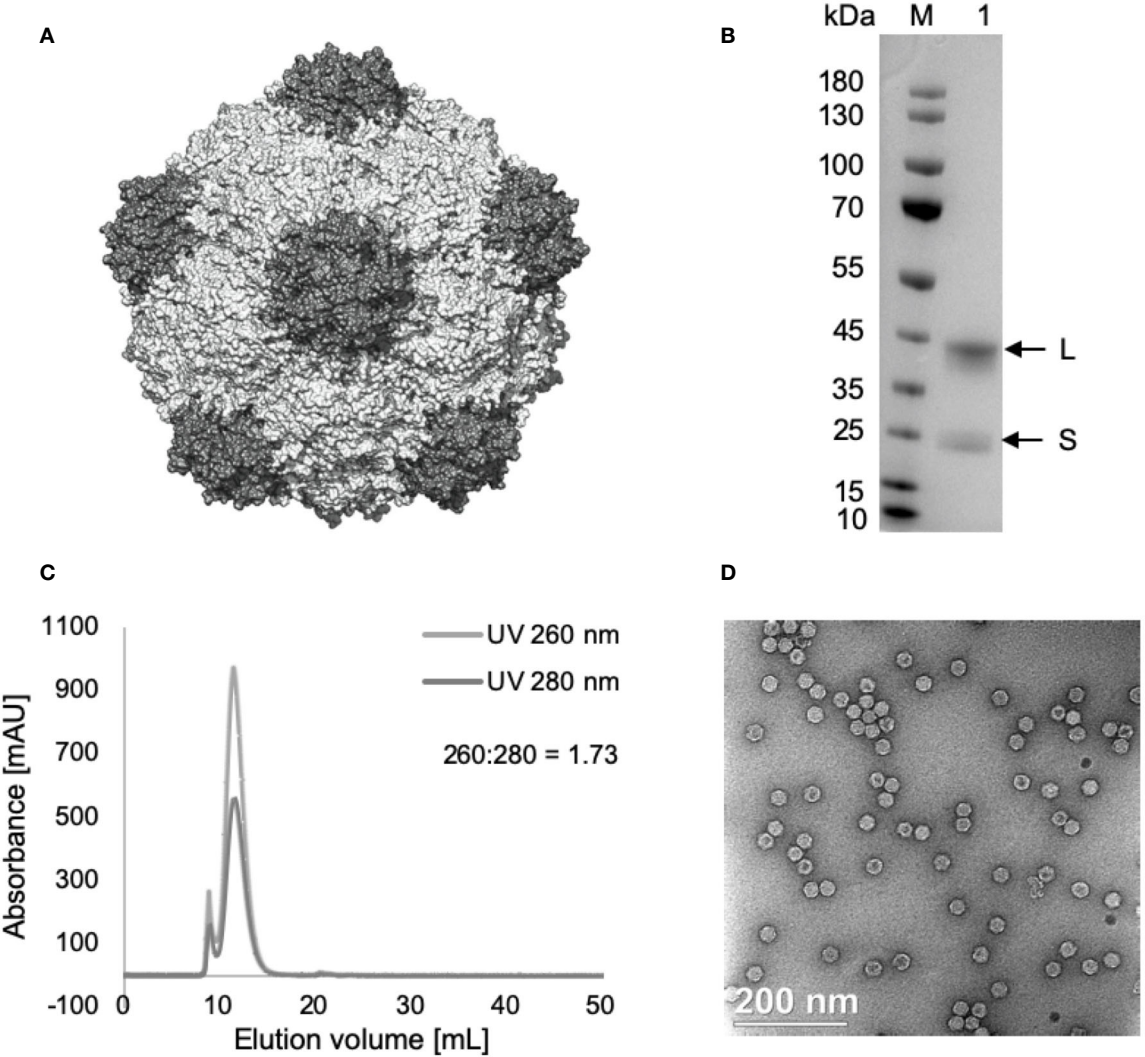
Cowpea mosaic virus (CPMV) particle structure and characterization. **(A)** Structure of CPMV, created using UCSF Chimera, using PDB entry 1ny7. **(B)** SDS-PAGE, 4%–12%, 200 V, 40’. Lane M: PageRuler Prestained Protein Ladder, lane 1: 5 µg CPMV. L (42 kDa) and S (24 kDa) coat proteins marked by arrows. **(C)** Size-exclusion chromatography of purified CPMV using Superose 6 10/300 column on GE Äkta^™^ Pure. **(D)** Transmission electron micrographs of uranyl acetate stained CPMV, 35,000x enlargement.

CPMV particles were propagated in the legume black-eyed pea plant No. 5 (*Vigna unguiculata* subsp. *Unguiculate*). Once propagated, the viruses were harvested, purified, and characterized as previously described (11) and demonstrated in **Figure 1**. Briefly, the plants were dusted with carborundum, an abrasive substance, and mechanically inoculated with 0.05 mg CPMV per leaf. Infected leaves were then harvested (10–15 days post inoculation), and the virus was purified by chloroform-butanol extraction, PEG precipitation, and sucrose gradient ultracentrifugation, as described previously (11). CPMV was characterized by SDS-PAGE, size exclusion chromatography using a Superose 6 column and fast liquid protein chromatography, as well as transmission electron microscopy of UAc-stained CPMV (**Figure 1**).Tumors were treated with a single injection of 100 μg CPMV intratumorally.

### Radiation

A Varian 2100 CD Linear Accelerator was used to deliver a uniform tumor dose of 15 Gy or 8 Gy to anesthetized mice. The beam was a 6 MeV electron beam with an SSD of 100 cm, encompassing the entire tumor, and a 2 mm peritumor region. The 3 x 8 Gy treatment was given on consecutive days.

### Immunohistochemistry

Following humane sacrifice, tumors were removed and sectioned. Previous studies have demonstrated the ability of this technique to accurately address morphological heterogeneity of the tumor. Following routine 4% buffered formaldehyde fixation and histological processing slides were stained with a CD8+ antibody (Cell Signaling Technology) according to manufacturer directions. Use of a methylene blue counterstain allowed for detailed identification of nuclei and the tagged CD8 membrane protein (stained black using nickel chloride). Stained and unstained cells were quantified using 20 randomly determine fields per slide at 40 X magnification. The result was expressed as the percentage of CD8+ vs total cell number.

### Protein Isolation and Quantification

Protein was isolated from tumors through homogenization in lysate buffer. Normalized samples were created, diluted in Tris-Buffered Saline. Western blots were performed using SDS-Page, with 3% bovine serum albumin used for blocking. Primary antibodies were incubated overnight, followed by an hour incubation in secondary antibody. A G-box Syngene was used for visualization of protein bands. HSP70, Pro-Caspase 3, and Cleaved Caspase 3 antibodies were used (Cell Signaling Technology). Protein bands were normalized to GAPDH expression.

### RNA Isolation and Quantification

RNA was isolated from tumors using the Qiagen RNeasy Mini Kit. Normalized samples were assayed for expression of 700+ genes quantified using NanoString PanCancer Immune Profiling Panel with additional apoptosis genes. The NanoString nCounter Analysis System was used according to the manufacturers protocol followed by assessment using the nCounter SPRINT system. Expression was quantified using the nSolver Analysis and Advanced Analysis Software.

### Statistical Methods

mRNA data was analyzed using NanoString nSolver Analysis and Advanced Analysis Software that utilizes XQuartz and R for statistical analysis. Differential expression significance is determined using the Benjamini-Yekutieli method to yield adjusted p-values, which allows for corrections due to multiple comparisons. For pathway expression scores and the immune cell abundance scores, ANOVA was used to determine statistical significance between the various treatment arms. For the Kaplan – Meier curve, GraphPad Prism was used for statistical analysis using Log-Rank (Mantel-Cox) test, with p <.05 indicating significance. For multiple comparisons of each survival curve, an adjust Bonferroni p-value of significance was set at p<.0033, based on the number of comparisons (15 if comparing all curves).

## Results

**Figure 2:**
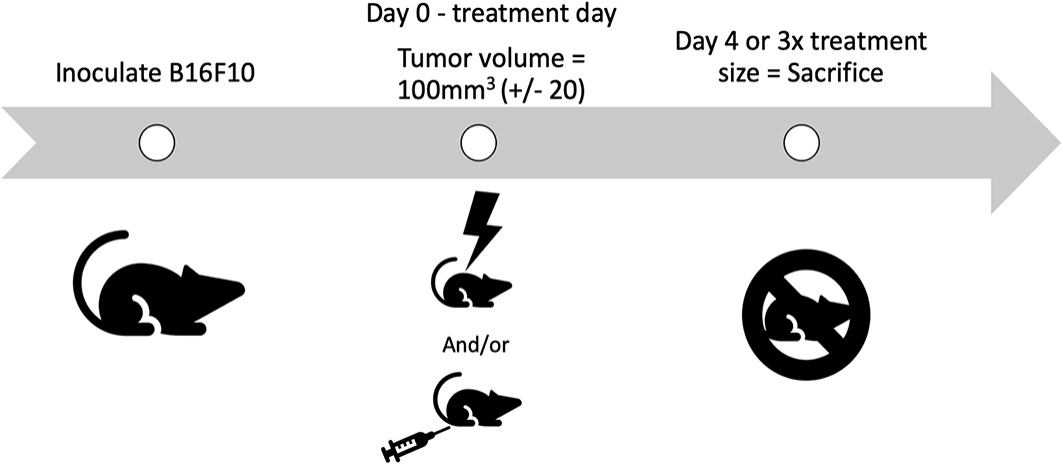
Schematic of the study design. B16-F10 cells are inoculated intradermally, with animals placed on study on day 0, when their tumors reach treatment size. Animals receive their treatment on day 0, either PBS, radiation, Cowpea mosaic virus (CPMV), or the combination. Two cohorts of animals are used for each treatment. One cohort is removed on day 4, for molecular and cellular investigation (n = 4–6), and the other cohort is removed once the tumors reach 3x the volume when treated (n = 4–5).

In this study, there were two cohorts of mice for each treatment. One cohort was taken off study four days post treatment, for molecular and cellular analysis of tumors. The other cohort was removed from the study when the tumor grew to 3x the volume it was on day 0. Day 0 is defined as the day animals are placed on study, when they receive treatment. CPMV was delivered on the first day of treatment intratumorally. A schematic illustrating the study design is depicted in **Figure 2**. It is important to note that none of the animals in this study experienced clinical morbidities or toxicities.

### RNA Expression of Immune & Cytotoxic Pathways

**Figure 3:**
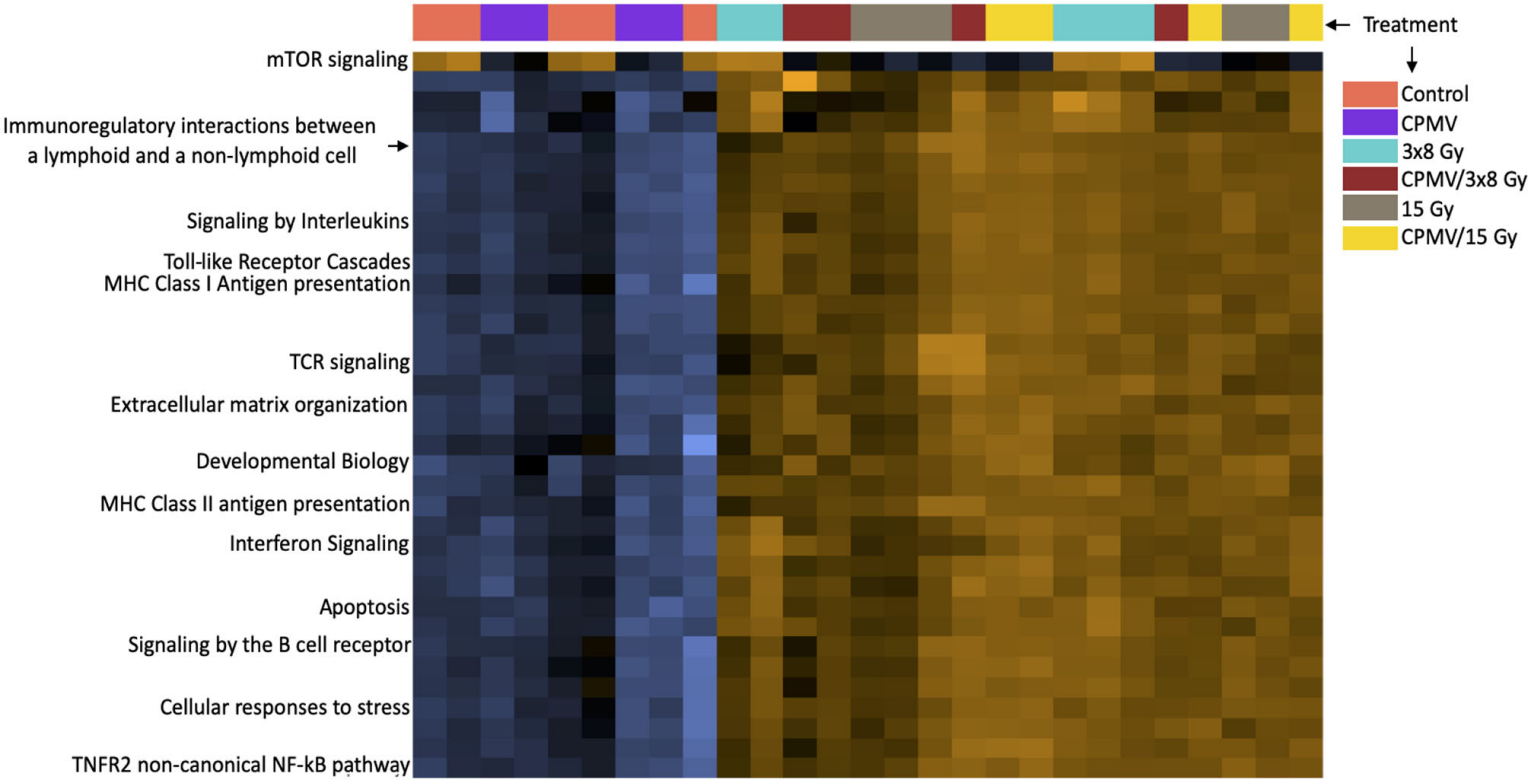
Heatmap of Pathway Expression. Pathway scores are derived from the first principal component of the genes in that pathways normalized expression, and range from -7.7 to 4.5. The NanoString analysis performs a Z-transformation to allow for scores to be displayed on the same scale, with yellow demonstrating high scores with blue demonstrating low scores. (i.e., yellow corresponds to most genes having higher expression in that pathway). The heatmap functions as a simplified way of seeing how treatment cohorts affect pathways differently, with each column representing an animal. While there are many pathways represented, we have labeled the ones we believe to be of importance such as the T cell receptor signaling pathway, antigen presentation pathways, etc. Many of these pathways demonstrate higher expression n scores in the combinatorial groups than either treatment alone. This is a high-level overview depiction of the mRNA results. This information is not appropriate for statistical analysis, as it is meant to be qualitative, not quantitative.

The nanoString cancer-immune gene panel included 770 genes and their associated pathways. The primary goal for this study was to determine the relative expression changes in important immune and cytotoxic genes and associated pathways following treatment with CPMV, two different hypofractionated radiation doses, and in combination. The notable changes in pathways reported below are summarized in the **Figure 3** heatmap. The most significant pathways are identified/labeled. These results are further synthesized in **Table 1**, with differential expression used to compare treatment and control cohort gene expression.

**Table 1:** Differential Expression. mRNA differential expression linear fold changes (standard error), as compared to control, for genes across the important cytotoxic and immune stimulatory pathways.

Gene	CPMV	3x8 Gy	15 Gy	3x8 Gy + CPMV	15 Gy + CPMV	Pathway/Description
**TLR1**	1.96* (1.3)	6.18* (1.2)	6.33* (1.2)	5.94* (1.2)	3.13* (1.2)	Toll-like receptor signaling pathway
**TLR 7/8**	2.5* (1.5)/1.03* (1.3)	9.64* (1.4)/5.64* (1.2)	16.31* (1.4)/5.96* (1.2)	18.4*^+^ (1.4)/6.45* (1.2)	21.2* (1.4)/7.28* (1.2)	Toll-like receptor signaling pathway
**CD80**	2.41* (1.4)	9.64* (1.4)	16.4* (1.4)	12.5* (1.4)	9.11*^+^ (1.4)	Toll-like receptor signaling pathway; Costimulatory molecule that stimulates T cells
**Jak1/2/3**	.866 (1.1)/1.05 (1.1)/.455* (1.3)	1.83* (1.1)/1.97* (1.1)/3.79* (1.2)	1.55* (1.1)/1.97* (1.1)/2.25* (1.2)	1.39*^+^ (1.1)/2.27* (1.1)/2.26*^+^ (1.2)	1.67* (1.1)/2.4*^+^ (1.1)/3.83*^+^ (1.2)	Jak-STAT signaling pathway, anti-apoptosis signaling
**Socs1/3**	.732 (1.3)/.441* (1.4)	6.42* (1.2)/1.94* (1.4)	3.28* (1.2)/.943 (1.4)	3.93*^+^ (1.2)/.676^+^ (1.4)	4.64* (1.2)/1.09 (1.4)	Jak-STAT signaling pathway, anti-apoptosis signaling
**Akt2**	.429* (1.1)	1.23* (1.1)	.532* (1.1)	.524*^+^ (1.1)	.505* (1.1)	PI3K-Akt and Jak-STAT signaling pathways, cell survival
**Tirap**	.156* (1.4)	.897 (1.4)	.18* (1.4)	.183*^+^ (1.4)	.227* (1.4)	Toll-like receptor signaling pathway, adaptor molecule in apoptotic cascade
**Irak1**	.493* (1.2)	1.23 (1.1)	.609* (1.1)	.43*^+^ (1.2)	.605* (1.1)	Toll-like receptor signaling pathway, chemotactic cascades
**Trif (ticam1)**	.395* (1.2)	1.42* (1.1)	.552* (1.2)	.425*^+^ (1.2)	.643* (1.2)	Toll-like receptor signaling pathway, chemotactic cascades
**CD247/CD3-zeta**	6* (1.9)	15.6* (1.8)	68.6* (1.8)	52.8*^+^ (1.8)	61.5* (1.8)	T cell receptor signaling pathway, T cell stimulation
**Gzmb**	.776 (1.6)	26.8* (1.4)	25.7* (1.4)	40.8* (1.5)	44.2* (1.5)	Immune mediated apoptosis pathway, pro-apoptosis
**Gzma**	1.13 (1.4)	24.7* (1.4)	11* (1.4)	80.6*^+^ (1.4)	69.4*^+^ (1.4)	Immune mediated apoptosis pathway, pro-apoptosis
**Trail/tnfsf10**	.603 (1.3)	4.74* (1.3)	4.14* (1.3)	2.77* (1.3)	4.1* (1.3)	Apoptosis pathway, pro-apoptosis
**Cd94/klrd1**	1.25 (1.4)	13.3* (1.3)	25.5* (1.3)	33.2*^+^ (1.4)	26.7* (1.4)	Natural Killer Cell mediated cytotoxicity pathway, activating receptor
**Icos**	2.08 (1.7)	16.1* (1.6)	24.7* (1.6)	21.5* (1.6)	23.9* (1.6)	T cell receptor signaling pathway, co-stimulator
**Ncr1 (nkp46)**	1.75 (1.9)	31.2* (1.6)	25.6* (1.6)	54.7*^+^ (1.6)	80.5*^+^ (1.6)	Natural Killer Cell mediated cytotoxicity pathway, activating receptor
**Nkg2c/klrc2**	1.25 (1.9)	15.4* (1.7)	15.4* (1.7)	24* (1.7)	33.2*^+^ (1.6)	Natural Killer Cell mediated cytotoxicity pathway, activating receptor
**Icam1/2**	1.55* (1.2)/.859 (1.2)	4.42* (1.1)/2.14* (1.2)	4.53* (1.1)/1.71* (1.2)	5.52*^+^ (1.1)/1.78* (1.2)	6.18*^+^ (1.1)/2.45*^+^ (1.2)	Natural Killer Cell mediated cytotoxicity pathway, binds to NK cells & activates
**CD86**	1.02 (1.3)	4.39* (1.2)	5.49* (1.2)	9*^+^ (1.2)	5.98* (1.2)	T cell receptor signaling pathway, T cell stimulation
**CD40**	2.5 (1.8)	26* (1.6)	44.8* (1.6)	42.3*^+^ (1.6)	38* (1.6)	T cell receptor signaling pathway, T cell stimulation
**NFkB (Nfkb1/nfkb2)**	.726* (1.1)/.917 (1.1)	1.44* (1.1)/2.09* (1.1)	1.15 (1.1)/1.66* (1.1)	.843^+^ (1.1)/1.75* (1.1)	1.99 (1.1)/2.44^+^ (1.1)	TNF signaling pathway, NFkB signaling pathway, cell survival

Changes in mRNA are reported for all treatment groups, with their importance demonstrated by the pathway/description and also described in the text. *indicates adjusted BY p <.05, as compared to control. ^+^indicates adjusted BY p <.05, as compared to the radiation alone (3x8 Gy or 15 Gy, respectively).

#### CPMV

One of the most significant gene pathway changes following CPMV was a decrease in the JAK-STAT pathway. This change predicts a decrease in anti-apoptotic signaling (increased tumor apoptosis), through down regulation of JAK, SOCS, and AKT (12–14). CPMV initiated increases in TLR1, TLR 7/8, and CD80, key players in the Toll-like Receptor Signaling Pathway. Increased expression indicates positive activation of immune stimulatory signals. There were however, also decreases in several genes, such as TIRAP, IRAK1, and TRIF, which might indicate CPMV also initiates a regulatory check in immune stimulatory activity (15, 16).

#### HFRT and HFRT + CPMV

In general, the HFRT alone and CPMV + HFRT treatments activated similar genes and pathways, however, in almost all cases, the combination resulted in a more therapeutically significant expression of the immune and cytotoxic genes and pathways. Numerous cytokines, cytokine receptors, chemokines, and cell adhesion molecules achieved greater upregulation. Both HFRT and HFRT + CPMV initiated a significant increase in up-regulation of the natural killer cell mediated cytotoxicity pathway, the toll-like receptor signaling pathway, the T cell receptor signaling pathway, and the apoptosis pathways. However, in almost all cases, the combination led to greater expression changes. In general expression increases in the associated genes, including CD80, CD94, Ncr1, Gzmb, and Gzma, that are directly associated with immune cytotoxicity cascades, both natural killer cell and T cell mediated cytotoxicity, was very significant. As was seen with CPMV alone, the combination treatment resulted in decreased expression of AKT, TIRAP, and TRIF and increased expression of TLR1, TLR7, and TLR8. Comparison of individual gene expression levels is presented in **Table 1**.

Compared to 15 Gy alone, CPMV + 15 Gy resulted in notable increases in the expression of genes associated with the natural killer cell mediated cytotoxicity pathway (Ncr1(NKp46), and NKG2C) and antigen processing/presentation to natural killer cells (ICAM1/2). Other significant expression increases, for CPMV + 15 Gy, were important factors in the T cell mediated apoptosis pathway (granzyme A and granzyme B) (17).

With respect to CPMV + 3x8 Gy (compared to 3x8 Gy alone), significant expression increases were seen for toll-like receptor and TNF signaling pathways, and natural killer cell mediated cytotoxicity pathways. The relative expression of most TLR genes decreased with 3x8 Gy + CPMV, however important genes associated with T cell stimulation (CD80, CD86, and CD40) were increased. NFkB, AKT, and other genes in the TNF signaling pathway were decreased indicating a decrease in cell survival signaling activity. Similar to the 15 Gy cohorts, genes such as Ncr1 and CD94 in the natural killer cell mediated cytotoxicity pathway, were upregulated indicating an increase in immune cell activation.

#### Differences Between 15 Gy and 3x8 Gy (Alone and in Combination With CPMV)

As mentioned previously, both HFRT doses resulted in significant anti-cancer genetic and biological activity, that was further enhanced by CPMV. There were however some differences between the two HFRT doses, both alone, and in combination. 15 Gy generated higher expression levels of ICOS, CD94, CD247, and CD80 (associated with T cell and natural killer cell cytotoxicity pathways). 3x8 Gy had higher expression of Ncr1, and Granzyme A, however there was no difference for Granzyme B or NKG2C. With the addition of CPMV, the same trends were true for ICOS, CD247, Granzyme A, and Granzyme B. 15 Gy + CPMV had greater activation of NKG2c and Ncr1 than 3x8 Gy + CPMV, while 3x8 Gy + CPMV demonstrated greater expression of CD94 and CD80.

Although subject to many variables, in an overall sense our data suggests 15 Gy alone and in combination stimulates a somewhat greater cytotoxic immune gene expression that does 3x8 Gy alone or in combination.

### Protein Expression Changes

The most interesting and potentially important protein expression changes, following CPMV + HFRT was the increased expression of the immunogenic heat shock protein 70 (HSP70), and the apoptotic “executioner” caspase 3. Expression of pro-caspase 3, the inactivate form of caspase 3, slightly decreased following CPMV and CPMV 3x8 Gy. Cleaved Caspase 3, the activated form of Caspase 3, was increased across all treatments with the greatest change seen in the HFRT and HFRT + CPMV cohorts. These protein levels, and corresponding mRNA expression levels, as compared to control, are demonstrated in **Table 2**.

**Table 2:** Protein & mRNA Expression. Protein & mRNA expression levels (as compared to control) for heat shock protein 70, caspase 3, and cleaved caspase 3.

Gene/protein	CPMV	15 Gy	CPMV 15 Gy	3x8 Gy	CPMV 3x8 Gy
**Hsp70 (hspa1b) mRNA**	1.04	1.27	1.08	.898	1.16
**HSP70 protein**	2.9	4.1	4.8	3.9	1.5
**Casp3 mRNA**	1.24	1.43	1.46	1.53	1.26
**Pro-caspase 3/Cleaved caspase 3 protein**	.74/2.2	1.1/17.2	1.2/33.5	1/23.2	.8/12.2

Changes in mRNA were also seen in protein, however were not closely correlated. As expected, changes in caspase 3 mRNA did not scale with the dramatic changes in cleaved caspase 3 protein. Unexpectedly, HSP70 mRNA did not correlate closely to the protein changes.

### Immune Infiltration

**Figure 4:**
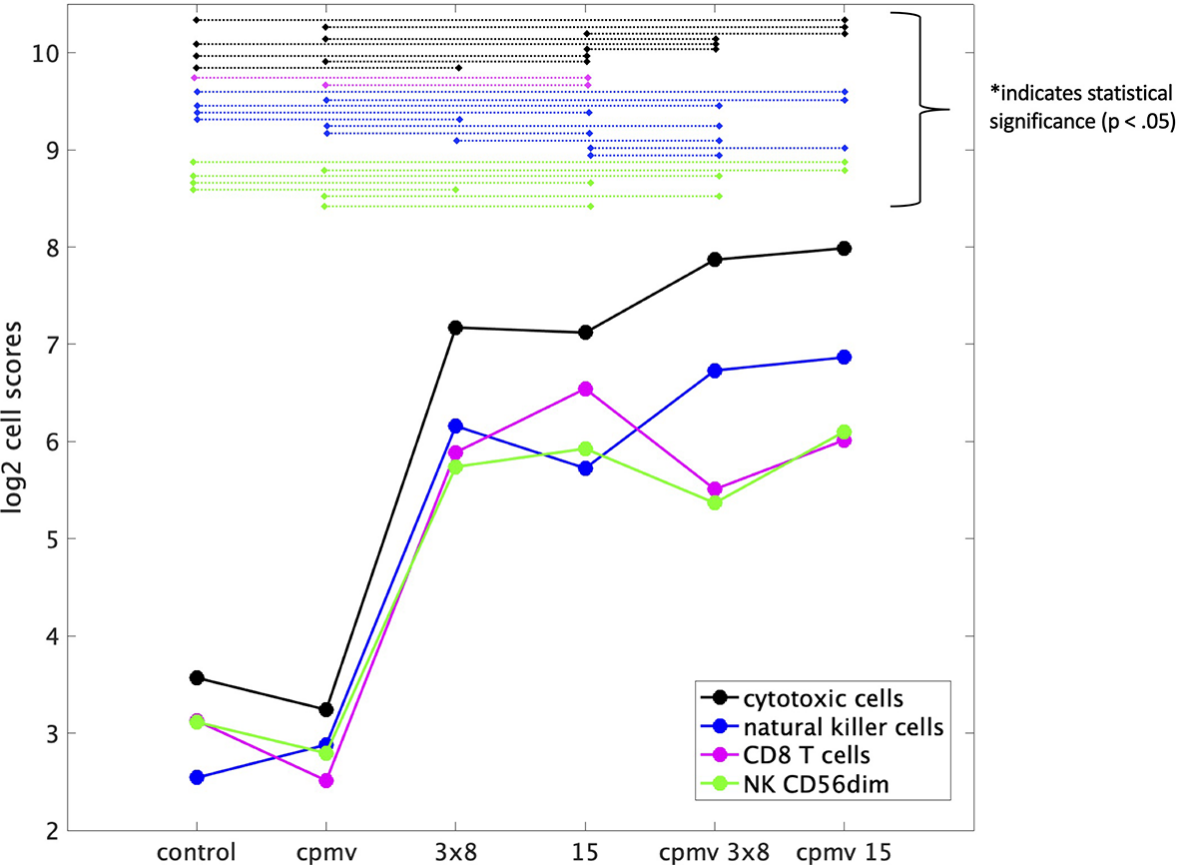
Immune Cell Abundances (mRNA). Cell type scores calculated using reference genes specific to certain cell types, for a variety of immune cell populations versus treatment cohorts. These scores (in the log_2_ space) represent cell abundance and allow for comparison of abundance across various treatments. Both cytotoxic and natural killer cells appear to have an increase of about 1 on the cell type score from 15 Gy to Cowpea mosaic virus (CPMV)/15 Gy indicating a two-fold increase in these cell populations. There is also an increase from 3x8 Gy to CPMV/3x8 Gy, but it is not as large as that between the 15 Gy cohorts. Statistical significance between groups is indicated by the dashed lines connecting the two treatment groups, as labeled.

Immune infiltration into tumors was assessed *via* RNA expression of genes characteristic to specific immune cell populations and by immunohistochemistry. NanoString analysis allows quantitation demonstration of various immune cell abundances, such as T cells, natural killer cells, and their activated populations (CD8 and CD56dim, respectively). The mRNA analysis showed that all treatments other than CPMV alone increased cytotoxic cells, CD8+ T cells, NK cells, and CD56dim NK cells. HFRT + CPMV increased the infiltration of natural killer cells (NK cells) and cytotoxic T cells (CD8) at least 2-fold as compared to any of the individual modalities (**Figure 4**). More specifically 15 Gy + CPMV appears to be somewhat more potent than 3x8 Gy + CPMV regarding recruitment of cytotoxic NK cells (NK CD56dim) and T cells (CD8).

Immunohistochemistry assessment demonstrated that all treatment groups had increased cytotoxic T cell (CD8+) infiltration of the tumors compared to the control. Approximately 1% of the total number of cells in a control/untreated tumor were cytotoxic/CD8+ cells. CPMV resulted in a 4-fold increase in CD8+ cells. A single radiation dose, 15 Gy and 15 Gy + CPMV treatment increased the CD8+ infiltration by17-fold. whereas 3x8 Gy and CPMV + 3x8 Gy increased CD8+ infiltration by approximately 10-fold. This data is detailed in **Table 3**. Statistical analysis, using an unbalanced two-way ANOVA, showed 15 Gy and 15 Gy + CPMV versus control achieved statistical significance (p<0.05).

**Table 3:** CD8+ Immune Infiltration. The average CD8+ populations for each treatment type with the standard deviations also reported.

Treatment	%CD8+ cells within the tumor (SEM)
Control	1.04 (0.34)
CPMV	4.23 (1.97)
3x8 Gy	9.56 (2.55)
15 Gy	17.71 (3.33)*
3x8 Gy + CPMV	10.32 (3.72)
15 Gy + CPMV	16.97 (4.65)*

Cowpea mosaic virus (CPMV) led to a mild increase in average population, with the radiation cohorts leading to a dramatic difference in average. However, the standard deviations were also dramatically increased, preventing statistical significance in many cohorts.

### Tumor Control

**Figure 5:**
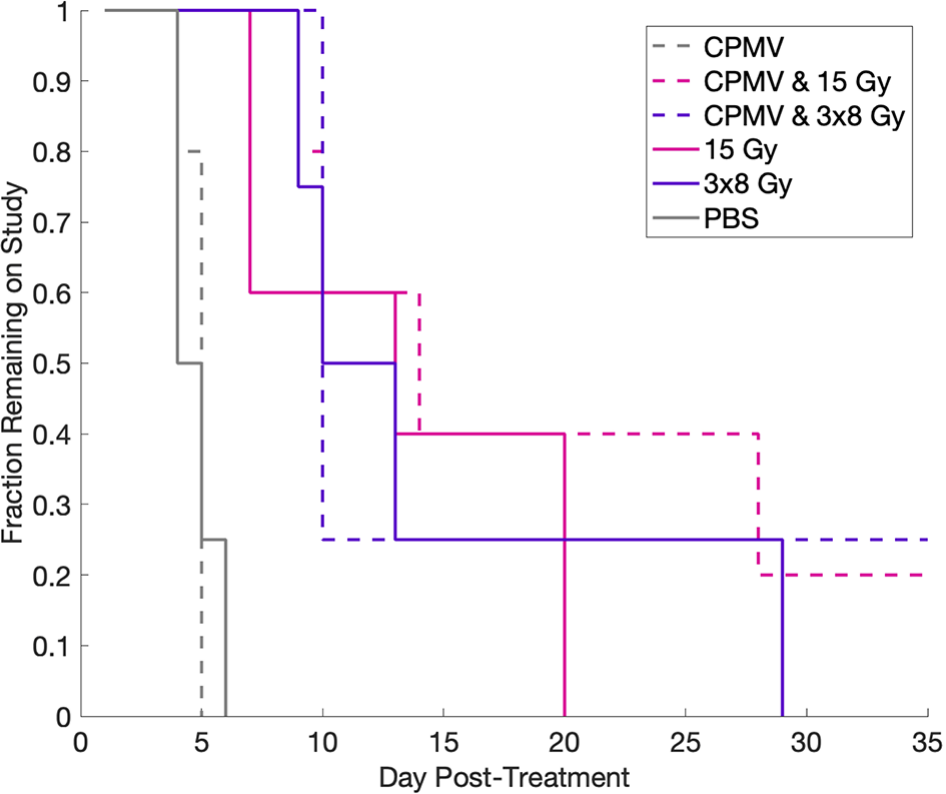
Kaplan-Meier Curve. Kaplan-Meier curve demonstrating the changes in fraction on study for each treatment as a function of days post-treatment. Control and Cowpea mosaic virus (CPMV) cohorts lack separation, whereas all of the radiation cohorts are separated from control and CPMV, but not from each other. Using the log-rank (Mantel-Cox) test, we determined that treatment did significantly change survival, however, using multiple comparison adjustment and comparing two curves to each other, only control versus 15 Gy and control versus 15 Gy + CPMV were statistically significantly different.

**Figure 6:**
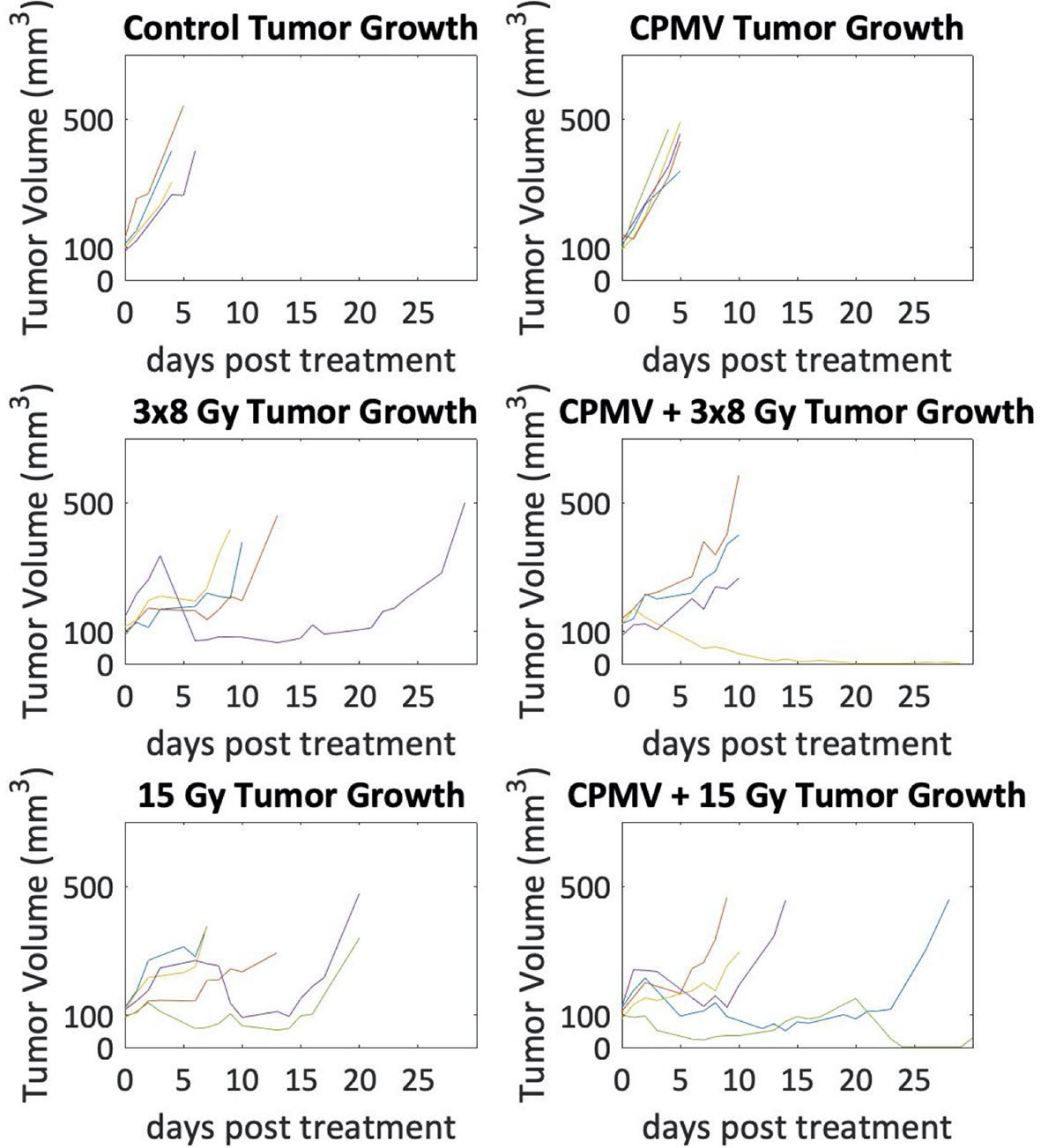
Tumor Growth Curves. Tumor growth curves for each animal with tumor volume on a logarithmic scale. Control and Cowpea mosaic virus (CPMV) tumor growth are exponential as the lines appear linear, whereas in the radiation cohorts there are several animals that do not follow exponential growth. Additionally, in the combinatorial CPMV and radiation cohorts there were a few animals whose tumor volumes decreased towards zero.

Tumor control efficacy was determined by assessing the number of days post treatment each animal was on study (3x tumor regrowth time, **Figure 5**). PBS treated tumors (control) averaged 4.75 days on study (SEM=0.48). HFRT alone, 15 Gy and 3x8 Gy, averaged 13.4 and 13.25 days, respectively (SEM=2.91, SEM=4.66). HFRT + CPMV resulted in regrowth periods of 14.5 days (SEM=6.5) and 19.4 days (SEM=5.36) for 3x8 Gy and 15 Gy, respectively. We performed the log-rank (Mantel-Cox) test on the survival curves altogether, and then also did pairwise comparisons. The p-value for comparison of all of the curves was <.0001 indicating the curves are significantly different. When completing the pairwise comparisons, an adjusted p-value was set for significance using a Bonferroni correction of p <.05/K where K is the number of comparisons, 15 in our case. The adjusted level of significance was p <.0033. Only two pair-wise comparisons satisfied this new criteria for statistical significance: control versus 15 Gy and control versus CPMV + 15 Gy. Two other pair-wise comparisons were close with p = .0035: CPMV versus 15 Gy and CPMV versus CPMV + 15 Gy. Tumor growth curves (**Figure 6**) demonstrate differences in the growth patterns for each treatment. CPMV + 15 Gy animals had the lowest growth rates, with several animals having a long period of tumor regression, as compared to the other treatment cohorts. As previous studies suggest, a single CPMV dose only had a modest effect on tumor control; multiple doses are necessary to notably affect tumor control, when used alone.

## Discussion

The primary experimental goal of this study was to assess the immune and cytotoxic response of CPMV combined with hypofractionated radiation therapy, with the hypothesis that the addition of CPMV would increase the cytotoxic immune response of HFRT, and that fraction regimens would matter. We chose a single intratumoral dose of 100 μg CPMV. Although this minimal dose regimen limits its immunogenic and biological efficacy potential (in previous work prime-boost treatments were applied (7, 18), it provided an experimentally meaningful preclinical therapeutic endpoint to assess in combination with radiation therapy. We acknowledge that a previous radiation + CPMV manuscript (8) shared two co-authors with this manuscript. The Patel study used a different cell line, a low radiation dose (10 Gy), multiple doses of CPMV that lacked RNA, and fewer methods of analyses (no mRNA expression or protein expression, and no quantitative IHC).

Overall, the results showed that even a modest single CPMV dose is capable of significantly enhancing the immune, cytotoxic and cellular anti-cancer responses caused by HFRT. Our data suggest a single dose of 15 Gy + CPMV has a somewhat greater immune and cytotoxic response than 3x8 Gy + CPMV. The data also shows the primary anti-cancer responses involve natural killer cell activation/cytotoxicity, as well as an increase in apoptotic activity, including T cell mediated apoptosis through cytotoxic T cell effector molecules. Specifically, immune effector genes include Granzyme A, Granzyme B, CD94, Ncr1, CD247, and NKG2C (19–21). As with many other immune modulators, it should be pointed out that both CPMV and HFRT stimulated, at the short post-treatment endpoint used, some genes that are conventionally believed to be in the pro-cancer category.

In this study, we attempted to validate, and where possible co-register, the immune and cytotoxic transcription changes with analysis of tumor protein, tumor cytotoxic T cell infiltration, and tumor control. Importantly all treatments led to increases in heat shock protein 70 (HSP70), which is a signal of immunogenic cell death and T cell mediated apoptosis, and cleaved Caspase 3, which is the last activating step leading to cell death by apoptosis (22, 23). Similar to the transcription studies, protein studies indicate that CPMV + 15 Gy activates the greatest increase in HSP70 and cleaved caspase 3. Overall, our post treatment RNA and protein results are closely correlated.

With respect to the post-treatment CD8+, tumor infiltration, even a single intratumoral dose of CPMV generated a 4-fold increase (compared to control). Significantly, 15 Gy and 15 Gy + CPMV increased that activity to > 16.5-fold. While our immunohistochemistry assessment was restricted to CD8+ T cells, the most clinically relevant immune cell type for most anticancer immune responses, the NanoString RNA data allows for quantitative analysis of several immune cell types. This validated significant immune and cytotoxic cell activation by HFRT and CPMV. Most notable, were the increases in activated cytotoxic natural killer cells (NK CD56dim) and activated cytotoxic T cells (CD8 T cells), as demonstrated by the mRNA expression data. The increase in cytotoxic natural killer cells and CD8 T cells indicates a greater cytotoxic immune response with the addition of CPMV to HFRT. The increased immune activation when combined with the inherent cytotoxic radiation effects leads to an overall better therapeutic potential and effect. One limitation of this study, in regards to comparing radiation doses, however is the timing differences of samples taken due to one dose being multiple fractions. However, we would expect this difference to lead to higher populations of immune cells in the fractionated regimen as more time is allowed for recruitment and infiltration. Based on experience in our lab, and comparisons with control, we do not believe the consecutive days of irradiation leads to drastic changes in immune cell populations in regards to potentially irradiating and killing immune cells within the tumor. Similar to our other results, these results translated into tumor control, and decreased tumor growth rates. The tumor growth study had an n=4/5 for each treatment arm. It is possible that increasing these animal numbers would lead to statistically significant differences between more of the treatment arms, however, based on the results from this study, we believe the animal numbers would have to be more than doubled to ever reach statistical significance between the four radiation groups.

### Conclusion

The most important finding in the study, using genetic, protein, immune cell tumor infiltration, and tumor control assays, was that a single intratumoral dose of an immunogenic plant virus was able to enhance the immune and cytotoxic effects of HFRT. Although adding a small dose of CPMV to HFRT did not translate into dramatic tumor control improvement compared to HFRT alone, the results indicate that the optimization of CPMV dosing with hypofractionated radiation could lead to a significant improvement in anti-tumor effect. Our molecular and cellular results indicate 15 Gy could be slightly more effective than 3x8 Gy when combined with CPMV, however, the difference in tumor growth was not significant.

## Data Availability Statement

The raw data supporting the conclusions of this article will be made available by the authors, without undue reservation.

## Ethics Statement

The animal study was reviewed and approved by Dartmouth IACUC.

## Author Contributions

KD: Experiment execution and analysis, manuscript initiation. RW: Experiment execution and analysis. VB: CPMV production/purification/analysis. SF: CPMV collaboration and direction. NS: CPMV collaboration and direction. PH: Scientific oversight and organization (PI). All authors contributed to the article and approved the submitted version.

## Funding

NCI U01 CA218292.

## Conflict of Interest

NS and SF are co-founders of and have a financial interest in Mosaic ImmunoEngineering Inc.

The remaining authors declare that the research was conducted in the absence of any commercial or financial relationships that could be construed as a potential conflict of interest.

## Supplementary Material

Supplementary Table 1 The genes included in the NanoString panel utilized in these experiments. This information is downloaded from the NanoString website.

Supplementary Table 2 Raw mRNA expression counts for genes that were assessed for all samples analyzed.

Supplementary Figure 1 Raw western blot images including the proteins discussed and highlighted in the manuscript.
