# Supplementary figures and images for: Cowpea Mosaic Virus Nanoparticle Enhancement of Hypofractionated Radiation in a B16 Murine Melanoma Model
Source: Front Oncol. 2020 Dec 16;10:594614. doi: 10.3389/fonc.2020.594614 (PMC7773968; doi:10.3389/fonc.2020.594614)

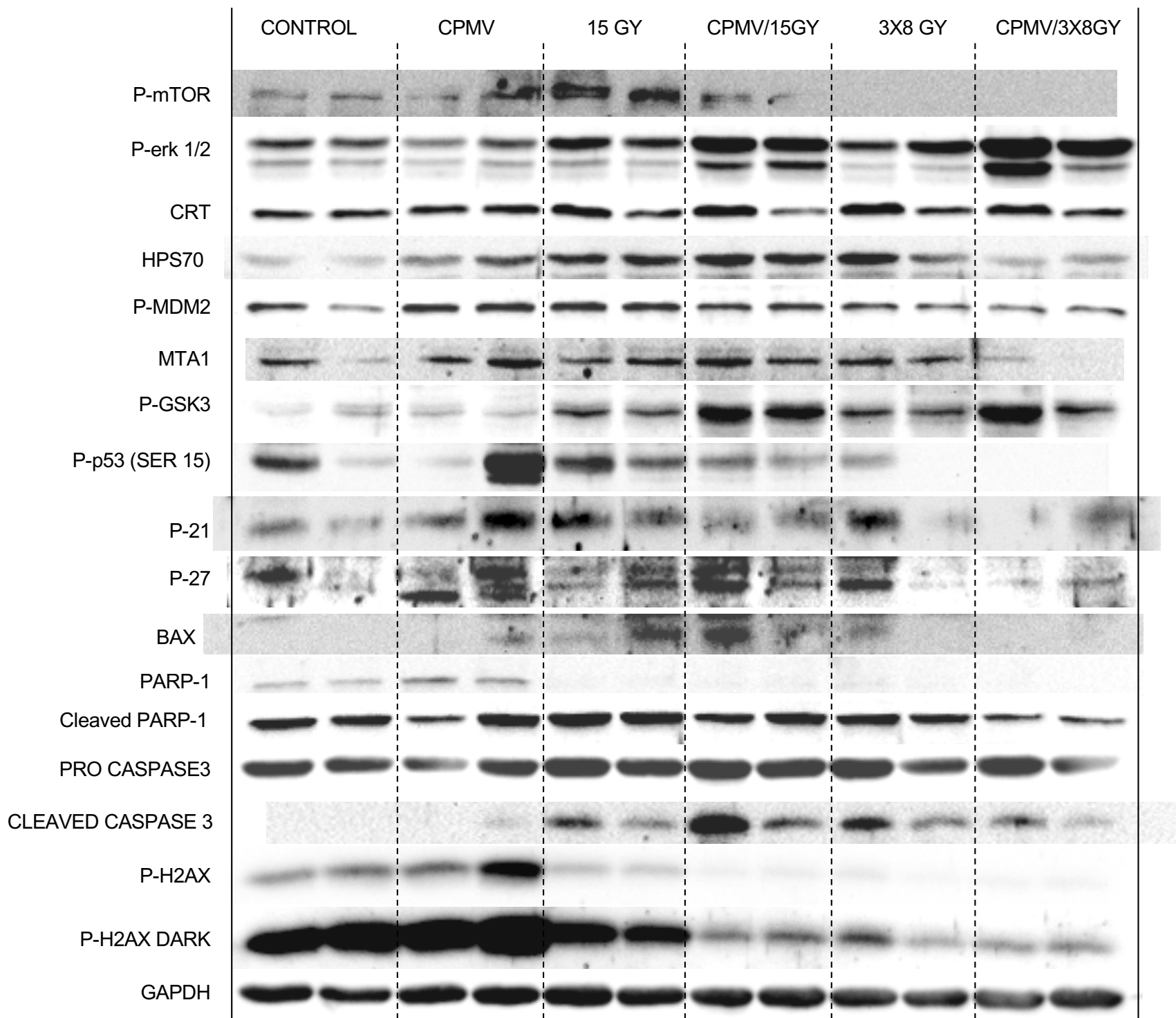

Supplement: Supplementary Figure 1 — Raw western blot images including the proteins discussed and highlighted in the manuscript. [file DataSheet_1.pdf]
